# Liraglutide, a human glucagon‐like peptide‐1 analogue, stimulates AKT‐dependent survival signalling and inhibits pancreatic β‐cell apoptosis

**DOI:** 10.1111/jcmm.13259

**Published:** 2018-03-10

**Authors:** Katerina Kapodistria, Effie‐Photini Tsilibary, Eleni Kotsopoulou, Petros Moustardas, Paraskevi Kitsiou

**Affiliations:** ^1^ Institute of Biosciences and Applications National Centre for Scientific Research, N.C.S.R. “Demokritos” Terma Patriarchou Grigoriou & Neapoleos Attiki Greece; ^2^ Center for Clinical, Experimental Surgery and Translational Research Biomedical Research Foundation Academy of Athens (BRFAA) Athens Greece

**Keywords:** Liraglutide, Pancreatic β‐cells, db/db diabetic mouse, Nephrin, PI3K‐AKT survival signalling, BAD inactivation, FoxO1 inhibition, apoptosis

## Abstract

Liraglutide, a human long‐lasting GLP‐1 analogue, is currently regarded as a powerful treatment option for type 2 diabetes. Apart from glucoregulatory and insulinotropic actions, liraglutide increases β‐cell mass through stimulation of β‐cell proliferation and islet neogenesis, as well as inhibition of β‐cell apoptosis. However, the underline molecular mechanisms have not been fully characterized. In this study, we investigated the mechanism by which liraglutide preserves islet β‐cells in an animal model of overt diabetes, the obese db/db mice, and protects a mouse pancreatic β‐cell line (βTC‐6 cells) against apoptosis. Treatment of 12‐week‐old diabetic mice with liraglutide for 2 weeks had no appreciable effects on blood non‐fasting glucose concentration, islet insulin content and body weight. However, morphological and biochemical examination of diabetic mouse pancreatic islets demonstrated that liraglutide restores islet size, reduces islet β‐cell apoptosis and improves nephrin expression, a protein involved in β‐cell survival signalling. Our results indicated that liraglutide protects βTC‐6 cells from serum withdrawal‐induced apoptosis through inhibition of caspase‐3 activation. The molecular mechanism of the anti‐apoptotic action of liraglutide in βTC‐6‐cells comprises stimulation of PI3‐kinase‐dependent AKT phosphorylation leading to the phosphorylation, hence inactivation of the pro‐apoptotic protein BAD and inhibition of FoxO1 transcription factor. In conclusion, we provided evidence that the GLP‐1 analogue liraglutide exerts important beneficial effects on pancreatic islet architecture and β‐cell survival by protecting cells against apoptosis. These findings extend our understanding of the actions of liraglutide and further support the use of GLP‐1R agonists in the treatment of patients with type 2 diabetes.

## Introduction

Type 2 diabetes mellitus (T2DM) is a progressive metabolic disease characterized by chronic hyperglycaemia. Pancreatic β‐cell dysfunction and insulin resistance of peripheral tissues contribute to its development 1. In the diabetic state, hyperglycaemia becomes apparent as insulin biosynthesis and secretion is progressively decreased and pancreatic β‐cell function gradually deteriorates 1, 2. Diabetic patients exhibit an early defect in glucose stimulated insulin secretion (GSIS) 3, 4. Also, β‐cell mass in T2DM patients declines progressively along the duration of the disease 2, 5. Such decline begins before the onset of T2DM 2, indicating that decreased β‐cell mass precedes chronic hyperglycaemia. It is widely accepted that long‐term strict glycaemic control preserves β‐cell function and mass against ‘glucose toxicity’ thus preventing from diabetic complications 6, 7. Accordingly, strategies for the treatment of TDM2 have been focused on agents that restore or preserve both β‐cell mass and function.

Recently, incretin‐based therapies associated with glucoincretin glucagon‐like peptide‐1 (GLP‐1) have been widely used for the treatment of T2DM 8. The incretin hormone GLP‐1, a glucose‐lowering polypeptide, has several beneficial effects that counteract the pathophysiology of diabetes mellitus. Pleiotropic effects of GLP‐1 include induction of glucose‐dependent insulin secretion 9, 10, suppression of glucagon secretion 11 and appetite suppression 12, 13. At the level of cell kinetics, GLP‐1 stimulates β‐cell replication 14, 15 neogenesis and differentiation 16, as well as inhibits β‐cell apoptosis *via* reduction of cellular stress 17, 18.

Liraglutide, a long‐acting human dipeptidyl peptidase‐4 (DPP‐4)‐resistant GLP‐1 analogue, appears to be a promising antidiabetic agent 19, 20. The substitution of Lys for Arg34 and the addition of a glutamic acid and a 16C NEFA to the Lys26 residue of native GLP‐1 help delay absorption and degradation of liraglutide by DPP‐4 21, 22. Liraglutide exerts its effect through the GLP‐1 receptor and effectively mimics the actions of mature GLP‐1, thus improving insulin secretion and insulin response while reducing insulin resistance 21, 23. Apart from glucoregulatory and insulinotropic actions, liraglutide increases β‐cell mass *in vitro* through stimulation of β‐cell proliferation and islet neogenesis, as well as inhibition of β‐cell apoptosis 18, 24. Nevertheless, the underlying molecular mechanisms have not been fully characterized.

In this study, we investigated the molecular mechanism by which liraglutide exerts its protective effects on survival of the cultured mouse pancreatic β‐cell line (βTC‐6 cells) as well as islet β‐cells of an animal model of overt T2DM, the db/db mouse. We provided evidence that treatment of diabetic mice with liraglutide restores islet size, reduces islet β‐cell apoptosis and enhances nephrin expression. Moreover, liraglutide stimulated AKT‐dependent survival signalling and suppressed apoptosis in βTC‐6 cells through inhibition of caspase‐3 activation. These findings extend our understanding of the anti‐apoptotic actions of liraglutide and further support the use of GLP‐1 analogues in the treatment of patients with type 2 diabetes.

## Materials and methods

### Antibodies and reagents

Goat polyclonal anti‐nephrin N20 (Cat. No: sc‐19000), rabbit polyclonal anti‐phospho‐Ser136 BAD (Cat. No: sc‐7999‐R), rabbit polyclonal anti‐BAD (Cat. No: sc‐942), antibodies were purchased from Santa Cruz Biotechnology, Inc. (Dallas, Texas, USA) Rabbit polyclonal anti‐AKT (Cat. No: 9272), rabbit monoclonal anti‐phospho‐Ser473 AKT (Cat. No: 40605), rabbit polyclonal anti‐phospho‐FoxO1(Thr24)/FoxO3a(Thr32) (Cat. No: 9464), rabbit polyclonal anti‐cleaved caspase‐3 (Cat. No: 9661) antibodies were purchased from Cell Signaling (Danvers, MA, USA). Mouse monoclonal anti‐insulin antibody (Cat. No: I 2018) was purchased from Sigma‐Aldrich (St. Louis, MO, USA). Fluorescent secondary antibodies, donkey anti‐goat Alexa Fluor 488 (Cat. No: A‐11055), donkey anti‐mouse Alexa Fluor 594 (Cat. No: A‐21203) and donkey anti‐mouse Alexa Fluor 488 (Cat. No: A‐21202) were purchased from Molecular Probes, Invitrogen (Thermo Fisher Scientific Waltham, MA, USA). The PI3K inhibitor, wortmannin (#9951), was purchased from Cell Signaling. Liraglutide (Victoza^®^) was a generous gift from Novo Nordisk Hellas (Agia Paraskevi, Attiki, Greece).

### Animals

The mouse model of type 2 diabetes BKS.Cg‐*m*+/+*Lepr* ^db^/BomTac was used *in vivo* studies 25. Ten‐week‐old male homozygous *(lepr−/−)* and heterozygous *(lepr+/−)* mice were purchased from Taconic Biosciences (New York, USA) and acclimatized for 2 weeks in the animal facility of the Biomedical Research Foundation of the Academy of Athens (BRFAA) under controlled ambient conditions (22°C, 45–55% humidity, 12:12‐hrs light/dark cycle with lights on at 07:00 am). Animals were given free access to drinking water and standard chow diet. On the twelfth week of age, the diabetic animals *(lepr−/−)* were divided into two groups (*n *=* *8 for each group) which were either treated with liraglutide (Novo Nordisk) (1000 μg/kg, ip, once daily) or vehicle (PBS ip, once daily) for 2 weeks. The respective control non‐diabetic animals *(lepr+/−)* were also divided into two groups (*n *=* *8), which were treated with liraglutide (1000 μg/kg, ip, once daily) or vehicle (PBS ip, once daily) for 2 weeks. The use of animal experimental protocols was approved by the local Ethics Committee. The dose of liraglutide used in the study was based on reviews of the literature 26. Bodyweight was monitored daily from 12 to 14 weeks of age. Non‐fasting blood glucose levels were monitored at the beginning of the treatment with liraglutide (the first day of 12 weeks of age) and at the end of the treatment (the last day of 14 weeks of age). At the end of treatment, animals were euthanized, pancreata were isolated and fixed in 10% neutral buffered formalin overnight and then embedded into paraffin wax.

### Cell line and culture conditions

Beta TC‐6 cells, a mouse T‐SV40 immortalized insulin secreting pancreatic beta‐cell line, (ATCC, Cat. No. CRL 11506) 27, were continuously grown in DMEM culture medium containing 1 mmol/l glucose (optimal glucose concentration for retention of glucose responsiveness and insulin secretion) 28, 15% (v/v) FCS, 4 mmol/l glutamine and antibiotics in 5% CO_2_ at 37°C. Medium was changed every 24 hrs, with fresh culture medium and cells subcultured as necessary to prevent over‐confluence. Cells were released from their tissue culture flasks for passaging by treatment with 0.25% (w/v) trypsin/0.03% (w/v) EDTA. For experiments, cells were used at passages 26–35.

### Activation of GLP‐1R signalling

GLP‐1R signalling was induced as follows: Cells cultured in 24‐well plates in the presence of 1 mmol/l glucose, were washed with PBS and incubated with Kreb's Hepes buffer (118.5 mmol/l NaCl, 2.54 mmol/l CaCl_2_2H_2_O, 1.19 mmol/l KH_2_PO_4_, 4.74 mmol/l KCl, 25 mmol/l NaHCO_2_, 1.19 mmol/l MgSO_4_7H_2_O, 10 mmol/l Hepes buffer, 0.1% (w/v) BSA, pH 7.4) for 60 min. (2 × 30 min.) at 37°C, to minimize phosphorylation of AKT induced by autocrine and paracrine‐insulin signalling. Cells were then stimulated with the appropriate concentration of liraglutide for 15–30 min. at 37°C. Finally, cells were washed with ice‐cold PBS, lysed and assessed using Western blotting analysis.

### *In vivo* analysis of nephrin‐fluorescence labelling of Formalin‐fixed Paraffin‐Embedded (FFPE) tissue

The well‐established mouse db/db model of diabetes was used to evaluate the effect of liraglutide on nephrin expression in *in vivo* studies. Dual immunofluorescence labelling of FFPE pancreatic tissue sections from control, diabetic and liraglutide‐treated mice, for nephrin and insulin was performed as previously described 29. Briefly, 3‐ to 4‐μm sections were cut from the embedded blocks and slides de‐waxed as follows: Twice in 100% xylene for 5 min., 100% ethanol for 10–20 sec., once in 90% ethanol for 10–20 sec. once in 70% ethanol for 10–20 sec. and twice in H_2_O for 10–20 sec. Antigen retrieval was performed in pre‐warmed (94–96°C) Dako target retrieval solution (S1699), for 30 min. in a water bath (at 95°C), 20 min. on the bench and 5 min. in running water. Slides were blocked with immunofluorescence buffer (IFF) (PBS plus 1% bovine serum albumin and 2% foetal calf serum) for 1 hr at RT. Slides were then incubated with primary antibodies diluted in IFF overnight at 4°C, washed (3 × 5 min. washes) in PBS and finally incubated with the appropriate fluorescent secondary antibodies (Molecular Probes) diluted in IFF for 1 hr at room temperature. Slides were washed with PBS, before mounting them with Dako Fluorescent Mounting Medium. Fluorescent specimens were examined with a confocal laser‐scanning microscope (Bio‐Rad, CA, USA). Images were obtained and processed with Adobe Photoshop CS4 version 11.0, software (Adobe Systems Incorporate, San Jose, CA, USA). Quantification of nephrin or insulin expression levels was performed in digital images. From each group (eight animals/group), 40 islets from random non‐coincident fields were captured. Nephrin or insulin fluorescence intensity was quantified in images using the Image J 1.43u image‐processing and analysis software (National Institutes of Health, Bethesda, Maryland, USA). Briefly, Image J was used to quantify the fluorescence intensity and the area of each islet using units of 8‐bit pixel brightness intensity values.

### *In situ* cell death detection TUNEL staining

Beta TC‐6 cells grown on glass coverslips were fixed with 4% (w/v) paraformaldehyde in PBS pH 7.4, for 1 hr at room temperature, permeabilized for 2 min. on ice with 0.1% TritonX‐100 in 0.1% sodium citrate and finally incubated with 50 μl TUNEL reaction mixture (TdT enzyme and fluorochrome labelling solution) for 1 hr at 37°C in the dark [*In Situ* Cell Death Detection Kit, TMR red (Roche Diagnostics, Mannheim, Germany)]. Finally, the cells were washed with PBS and incubated with DAPI (1 μg/ml) in PBS for 5 min., before mounting with Dako Fluorescent Mounting Medium (Cat. No: S3023, Dako, Glostrup, Denmark). Specimens were examined with a confocal laser‐scanning microscope (TCS SP5 Confocal system; Leica, Wetzlar, Germany). Images were obtained and processed with Adobe Photoshop CS4 version 11.0, software. Quantification of the percentage of cells undergoing apoptosis was performed in the acquired digital images.

The *In Situ* Cell Death Detection Kit, TMR red (Roche Diagnostics), was also used for the assessment of apoptosis in 3‐μm‐thick pancreatic sections derived from the db/db mice. Briefly, slides were de‐waxed, rehydrated and permeabilized with proteinase K solution (20 μg/ml) for 15 min. at RT. After washing, sections were incubated with 50 μl TUNEL reaction mixture (TdT enzyme and fluorochrome labelling solution) for 1 hr at 37°C in the dark. To visualize apoptotic β‐cells, after TUNEL labelling, sections were blocked with immunofluorescence buffer (IFF) (PBS plus 1% bovine serum albumin and 2% foetal calf serum) for 30 min. at RT and were then incubated with anti‐insulin antibody diluted in IFF, for 2 hrs at RT, followed by staining with the appropriate fluorescent secondary antibody (anti‐mouse Alexa Fluor 488; Molecular Probes). Finally, slides were washed with PBS, before mounting them with Dako Fluorescent Mounting Medium. Specimens were examined with a confocal laser‐scanning microscope (LEICA SP8‐MP). From each group (eight animals/group), 40 islets from random non‐coincident fields were captured. Quantification of the percentage of islets containing apoptotic β‐cells was performed in digital images that were acquired using Adobe Photoshop CS4 software and saved in a lossless format.

### Western blotting

For Western blotting, following appropriate treatment where applicable, cells were lysed in Hepes lysis buffer [100 mmol/l NaCl, 1% (v/v) Triton‐X‐100, 0.5% (w/v) sodium deoxycholate, 0.2% (w/v) SDS, 2 mmol/l Na_2_EDTA, 10 mmol/l Hepes buffer (pH 7.5), 1xPhosSTOP (cocktail of phosphatase inhibitors, Roche) and 1xProtease inhibitors cocktail (Roche, Mannheim, Germany). Protein concentration was determined by the Bradford colorimetric assay (Pierce, Thermo Fisher Scientific Waltham, MA, USA). Immunoblot analysis of cell lysates was performed as previously described 30. To normalize protein load amounts, the blots were stripped (Re‐Blot Plus Mild Solution, Cat. No: 2502, Millipore, Thermo Fisher Scientific Waltham, MA, USA) and re‐probed with anti‐β‐tubulin monoclonal antibody (Cat. No: T4026; Sigma‐Aldrich).

### Statistical analysis

Values are presented as means ± S.D. Statistically significant differences between values were evaluated by one‐way ANOVA. A *P* value <0.05 was considered statistically significant. Statistical analysis of the results from the sections of the db/db diabetic animals treated or not with liraglutide and their controls was also performed by Mann–Whitney nonparametric test.

## Results

### Metabolic variables in diabetic mice treated with liraglutide

**Figure 1:**
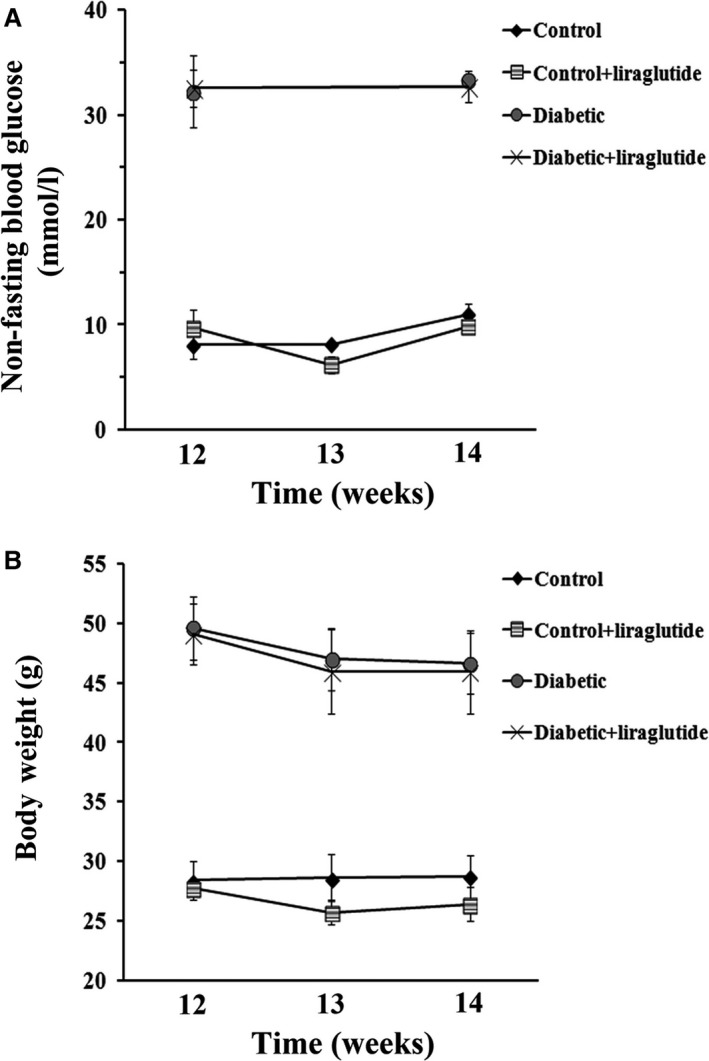
Metabolic variables of 12‐week‐old diabetic (*lepr−/−)* and control (*lepr+/−)* mice treated for 2 weeks with liraglutide or vehicle. (**A**) Non‐fasting blood glucose concentration. (**B**) Bodyweight. Data represents the mean ± S.D; *n* = 8 for each group. Comparisons between groups were performed using one‐way ANOVA and Mann–Whitney nonparametric test.

At the baseline, 12 weeks of age, blood glucose levels (mmol/l) and weight (g) were measured. Blood non‐fasting glucose levels rather than fasting glucose were monitored as they better reflect the overall pathophysiological process of diabetes, that is insulin resistance, inadequately suppressed hepatic glucose output and defective insulin response to meals 31. Control mice (*lepr+/−*) weighed 28.4 ± 1.63 g and showed blood non‐fasting glucose concentration of 8.06 ± 1.3 mmol/l. Diabetic mice (*lepr−/−*) with a weight of 49.6 ± 2.64 g showed pronounced hyperglycaemia (32.23 ± 3.41 mmol/l) (Fig. 1). After 2 weeks of treatment, liraglutide did not significantly affected blood non‐fasting glucose levels (32.64 ± 1.49 *versus* 33.31 ± 0.23 mmol/l) and bodyweight (45.9 ± 3.51 *versus* 46.6 ± 2.57 g) of diabetic mice compared with vehicle (PBS)‐treated mice (Fig. 1). Similarly, there were no significant differences in blood non‐fasting glucose levels and bodyweight among control animals (*lepr+/−*) treated or not with liraglutide (Fig. 1).

### Liraglutide restores nephrin expression and islet size in diabetic mice

**Figure 2:**
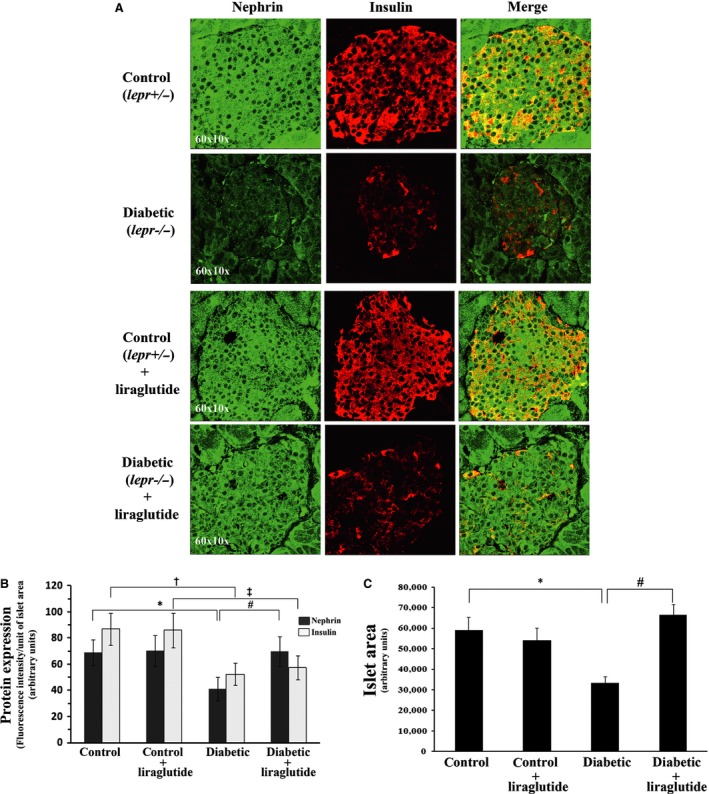
Liraglutide restores islet β‐cell nephrin expression and islet area in diabetic mice. Nephrin and insulin were detected by fluorescence immunohistochemistry in sections of formaln‐fixed paraffin‐embedded murine pancreata from db/db mice. (**A**) Representative confocal images of mouse pancreas sections from diabetic *(lepr−/−)* animals show reduced expression of both nephrin (green) and insulin (red) in the pancreatic islet compared with the islet of control (*lepr+/−)* animal. Conversely, islets of diabetic animals treated with liraglutide exhibited a significant increase in the levels of nephrin compared with islets of diabetic animals treated with vehicle (PBS). Liraglutide had no significant effect on nephrin expression in normoglycaemic animals and on islet insulin content in both normoglycaemic and diabetic animals. Magnification: ×600. Staining of mouse pancreata sections with insulin (red) and the secondary antibody anti‐goat IgG‐Alexa Fluor 488 (green) alone, gave no signal for nephrin (negative control) (data not shown). (**B**) Quantification of islet nephrin or islet insulin levels (protein fluorescence intensity/unit of islet area). Data represent the means ± S.D. from measurements of approximately 40 independent islets per group (five islets per mouse pancreas were randomly chosen from eight animals per group); *P <* *0.05 as compared to nephrin expression in islets of control animals; #*P *<* *0.05 as compared to nephrin expression in islets of diabetic animals; †*P *<* *0.05 as compared to islet insulin content in control animals; &*P *<* *0.05 as compared to islet insulin content in control animals treated with liraglutide. (**C**) Evaluation of islet area. Data represent the means ± S.E. from measurements of approximately 40 independent islets per group (five islets per mouse pancreas were randomly chosen from eight animals per group); *P <* *0.05 as compared to islet area in control islets; #*P *<* *0.05 as compared to islet area in diabetic islets.

We have previously shown a pivotal role for nephrin in pancreatic β‐cell survival through nephrin‐induced PI3K/AKT anti‐apoptotic signalling 32. Immunohistochemical studies in pancreatic islets of T2DM *(*db/db *lepr−/−)* mice revealed an *in vivo* reduction of nephrin expression which was accompanied by significantly decreased levels of phosphorylated AKT. The effect of liraglutide on nephrin expression in chronic hyperglycaemia was examined in the animal model of db/db mouse. These mice are leptin receptor deficient and a well‐established model of T2DM; they are obese, hyperinsulinemic and exhibit pronounced hyperglycaemia after the eighth wk of age 33, 34. Dual fluorescence immunohistochemical studies with nephrin and insulin in mouse pancreatic sections revealed that the islets of (*lepr−/−)* diabetic animals treated with liraglutide exhibited a significant increase in the levels of nephrin/per unit of islet area (69.89 ± 10.39) compared with islets of *lepr−/−* animals treated with PBS (41.23 ± 8.87) (Fig. 2A and B); the levels of nephrin expression in liraglutide‐treated diabetic animals (*lepr−/−)* were nearly the same (69.89 ± 10.39) as those in liraglutide treated (70.38 ± 11.73) or not treated (68.93 ± 9.76) control animals *(lepr+/−)* (Fig. 2A and B). Liraglutide had no effect in nephrin expression in control animals *(lepr+/−)*. Conversely, liraglutide did not manage to improve islet insulin content in diabetic animals. The islets of diabetic (*lepr−/−)* animals treated with liraglutide exhibited similar, low levels of insulin/per unit of islet area (57.34 ± 9.34) compared with islets of diabetic animals treated with PBS (52.32 ± 8.56) (Fig. 2A and B). Liraglutide had no effect in islet insulin content in control *(lepr+/−)* animals (86.02 ± 12.34) compared with islets of control animals treated with PBS (86.83 ± 12.13) (Fig. 2A and B).

In addition, examination of islets demonstrated that islet area of diabetic *(lepr−/−)* mice was markedly decreased (33380 ± 3083) (arbitrary units) compared to control animals (*lepr+/−)* (59108 ± 6205) (Fig. 2C). However, treatment of diabetic mice with liraglutide restored islet area (66361 ± 5341) (Fig. 2C). Liraglutide had no significant effect on the area of islets in control animals (54078 ± 6120) (Fig. 2C).

### Liraglutide reduces islet β‐cell apoptosis in diabetic mice

**Figure 3:**
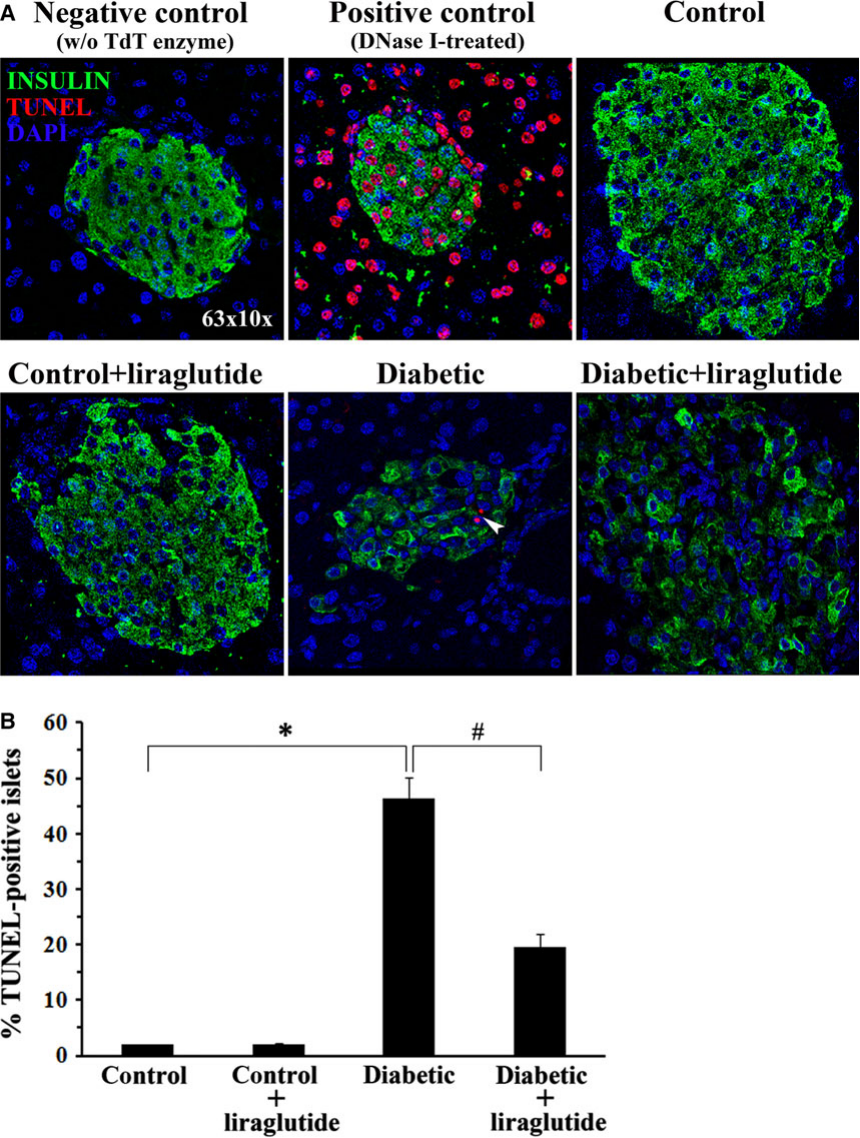
Liraglutide reduces islet β‐cell apoptosis in diabetic mice. Beta‐cell apoptosis was evaluated by *in situ* TUNEL and DAPI staining in sections of formalin‐fixed paraffin‐embedded murine pancreata from diabetic (*lepr−/−)* and normoglycaemic *(lepr+/−)* mice, treated or not with liraglutide (**A**) Representative confocal images of islets containing apoptotic β‐cells (white arrowhead). Magnification: ×630. DNase‐treated sections were used as a positive control in the TUNEL assay, whereas sections incubated with labelling reaction mixture in the absence of TdT enzyme served as a negative control. (**B**) Quantification of the percentage of islets containing TUNEL^+^ insulin^+^ cells. Data represent the means ± S.D. from measurements of approximately 40 independent islets per group (five islets per mouse pancreas were randomly chosen from eight animals per group); *P <* *0.05 as compared to islets in control animals; #*P *<* *0.05 as compared to islets in diabetic animals.

Assessment of apoptosis in pancreatic sections, demonstrated that the percentage of islets containing TUNEL^+^ Insulin^+^ cells was dramatically increased in diabetic animals *(lepr−/−)* compared with control animals *(lepr+/−)* (Fig. 3A and B). However, 2 weeks of treatment with liraglutide resulted in significant reduction of the percentage of islets containing apoptotic β‐cells (Fig. 3A and B). Taken together with the earlier results (Fig. 2A‐C), these data suggest that liraglutide increases and preserves β‐cell mass, in part by preventing apoptosis as well as improving nephrin expression and therefore nephrin‐mediated survival signalling.

### Liraglutide stimulates PI3K‐dependent AKT signalling in cultured β‐cells

**Figure 4:**
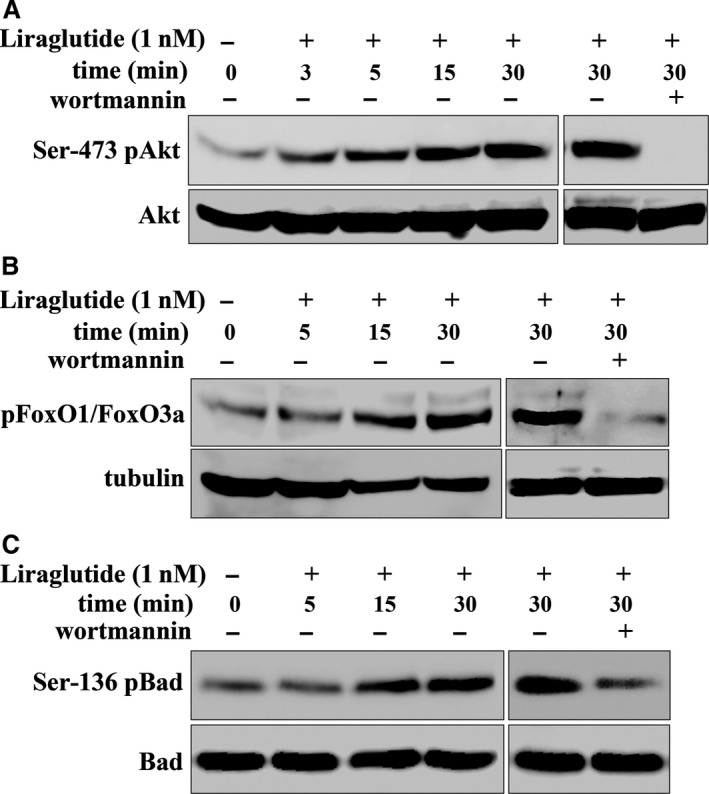
Liraglutide stimulates PI3K‐dependent AKT signalling in cultured pancreatic βTC‐6 cells. Beta TC‐6 cells grown on 24‐well plates were pre‐treated or not with the PI3K inhibitor, wortmannin for 1 hr and finally stimulated with 1 nmol/l of liraglutide for 3–30 min. Cells were then lysed and AKT‐, BAD‐ and FoxO‐phosphorylation were determined by immunoblotting. Three independent experiments were performed. (**A**) Representative Western blot of (Ser473) AKT phosphorylation. Immunoblots were stripped and re‐probed with anti‐AKT antibody to normalize the blots for protein levels. (**B, C**) Representative Western blots of (Thr24) FoxO1/(Thr32) FoxO3a and (Ser136) BAD phosphorylation. Immunoblots were stripped and re‐probed with anti‐tubulin or anti‐Bad antibody to normalize the blots for protein levels.

The mechanism of β‐cell‐protection by liraglutide was examined in the βTC‐6 cell line *in vitro*. We initially investigated whether liraglutide can trigger PI3 kinase‐dependent AKT signalling. Therefore, βTC‐6 cells were stimulated with 1 nmol/l of liraglutide and the phosphorylation pattern of selected proteins was examined. Figure 4A demonstrates that treatment of cells with liraglutide resulted in time‐dependent increased phosphorylation of the pro‐survival kinase AKT at Ser473, compared to untreated cells. Liraglutide‐induced AKT phosphorylation was completely inhibited by the PI3K inhibitor wortmannin, demonstrating that phosphorylation of AKT is PI3K dependent (Fig. 4A).

In order to confirm liraglutide‐mediated AKT activity, we monitored the extent of phosphorylation of BAD and FoxO1/FoxO3a (Forkhead box O family) transcription factors, both being pro‐apoptotic elements, whose activity is suppressed by AKT‐mediated phosphorylation at Ser 136 and Thr24/32, respectively 35. Western blotting analysis demonstrated that liraglutide also induced a time‐dependent significant increase in both BAD and FoxO phosphorylation, compared to untreated cells (Fig. 4B and C). Liraglutide‐induced BAD and FoxO suppression was completely inhibited by the PI3K inhibitor wortmannin demonstrating that their phosphorylation is PI3K dependent (Fig. 4B and C). Similar results were obtained when liraglutide was used at a final concentration of 10 nmol/l (data not shown). These results indicate that liraglutide triggers PI3K‐dependent AKT anti‐apoptotic signalling in β‐cells, in part *via* inhibition of BAD and FoxO1/FoxO3a activity.

### Liraglutide protects cultured β‐cells from serum withdrawal‐induced apoptosis

**Figure 5:**
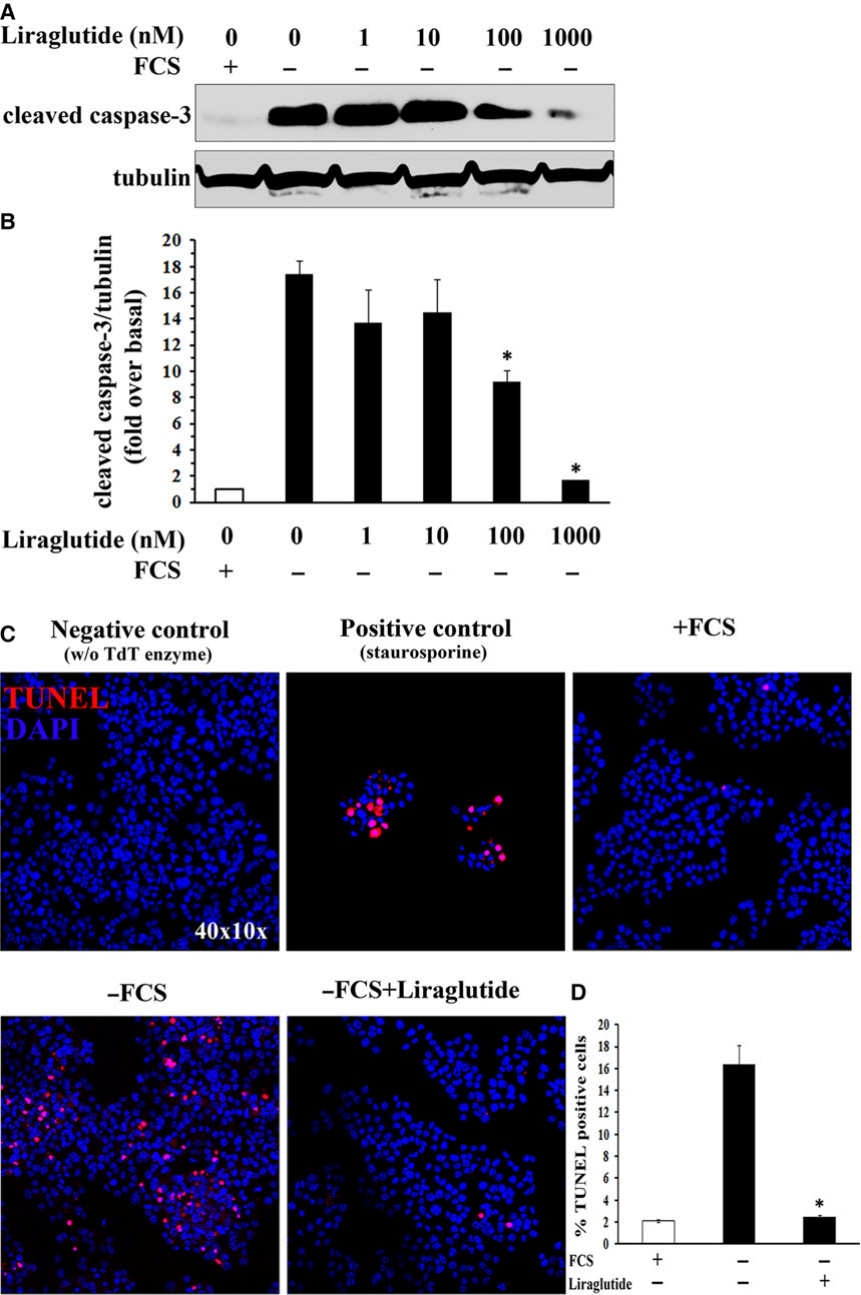
Liraglutide preserves cultured βTC‐6 cells from serum withdrawal‐induced apoptosis. Beta TC‐6 cells grown on 24‐well plates were incubated for 48 hrs in the absence of serum, in medium containing increasing concentrations of liraglutide. Cell apoptosis was assessed by detection of caspase‐3 activation (cleavage) and *in situ* TUNEL. (**A**) Representative Western blotting of cleaved (activated) caspase‐3. Immunoblots were stripped and re‐probed with anti‐tubulin antibody to normalize the blots for protein levels. (**B**) Densitometric quantification of activated caspase‐3 to tubulin levels as fold over basal rate of activated caspase‐3 (cells grown in the presence of serum, white bar). Data presented as the mean values ± S.D.; *n *=* *3, *P <* *0.05 as compared to activated caspase‐3/tubulin levels of serum depleted cells incubated in the absence of liraglutide. (**C**) Cell apoptosis was evaluated by *in situ* TUNEL and DAPI staining. Representative confocal images of apoptotic cells (red: *in situ* TUNEL; blue: DAPI). Magnification: ×400. Cells treated with 200 nmol/l of staurosporine for 18 hrs, served as positive control for induction of apoptosis, whereas cells incubated with labelling reaction mixture in the absence of TdT enzyme or cells growing in the presence of serum served as negative control. (**D**) Quantification of the percentage of cells undergoing apoptosis. In each case, a total of 1000–1500 cells, from three independent experiments, were used to evaluate the number of TUNEL‐positive cells. Data represent the means ± S.D.; *n *=* *3, *P <* *0.05 as compared to serum depleted cells incubated in the absence of liraglutide.

Next, we investigated the potential protective effect of liraglutide on cell survival. Beta TC‐6 cells were depleted from serum, incubated in the presence or absence of increasing concentrations of liraglutide (1–1000 nmol/l) for 48 hrs and finally lysed. Caspase‐3 activation was monitored by caspase processing. The amount of cleaved caspase‐3 has been previously correlated with pancreatic β‐cell apoptosis 32, 36. Western blotting analysis demonstrated that upon serum depletion, βTC‐6 cells displayed increased levels of the large fragment (19 kD) of activated caspase‐3 (17.45 ± 1.44) compared to cells grown in the presence of serum (1 ± 0.00) (Fig. 5A and B). However, activated caspase‐3 levels were reduced in the presence of 100 nmol/l of liraglutide (9.21 ± 1.19), whereas 1000 nmol/l of liraglutide effectively inhibited caspase‐3 activation (1.73 ± 0.001) (Fig. 5A and B).

Additionally, the degree of apoptosis was evaluated by detection of DNA strand breaks (*in situ* TUNEL and DAPI staining). Analysis of βTC‐6 cells for DNA fragmentation (TUNEL‐positive cells) revealed that serum deprivation resulted in an eightfold increase in the number of TUNEL‐positive cells, compared to cells grown in the presence of serum (where baseline apoptosis was minimal) (Fig. 5C and D). However, cell apoptosis was efficiently inhibited by 1000 nmol/l of liraglutide (Fig. 5C and D). Following 48 hrs of culture, both control and treated cells demonstrated similar levels of apoptosis. Taken together the above results suggest that liraglutide enhances β‐cell survival by inhibiting cell apoptosis.

## Discussion

New strategies for the treatment of T2DM have been focused on agents that restore and/or preserve β‐cell mass and eventually β‐cell functions. Liraglutide, a long‐lasting human GLP‐1 analogue, is currently being used for the treatment of patients with T2DM. Numerous studies have shown that drugs acting on the GLP‐1R axis (incretin‐related drugs) preserve both β‐cell function and mass in animal models of diabetes 26, 37, 38. Therefore, a better understanding of the protective mechanisms activated by GLP‐1 analogues in the islet β‐cell is potentially relevant to the therapy of the disease.

In the present study, the well‐established mouse model of T2DM, db/db mouse, was used to evaluate the effects of liraglutide on islet β‐cells in *in vivo* studies. We demonstrated that a 2‐week treatment with liraglutide had no significant effects on blood non‐fasting glucose concentration, islet insulin content and bodyweight in 12‐week‐old db/db mice. This might be surprising given the well‐documented effects of GLP‐1 receptor activation on glucose metabolism, satiety and weight loss in humans 8. However, it agrees with previous studies demonstrated that liraglutide had no appreciable effects on bodyweight, food intake, non‐fasting glucose and insulin concentrations in 10‐ to 12‐week‐old db/db mice with degenerative diabetes 39. Conversely, Shimoda *et al*. 2011 26 demonstrated an improvement of metabolic variables in younger, 9‐week‐old db/db mice, which were treated with liraglutide for 2 weeks. Given that the db/db mice exhibit a severe age‐related form of diabetes with significant deterioration of metabolic status as beta‐cell mass fails to be sustained 40, discrepancies regarding the response to liraglutide treatment could be attributed to age‐related differences in mice used in these studies. Indeed, it was reported that the beneficial effects of liraglutide on pancreatic β‐cell function were more powerful during the early stages of diabetes (7‐week‐old mice) compared to those in advanced stage (16‐week‐old mice) 41. It remains to be substantiated whether a longer treatment strategy with liraglutide or treatment with a combination of liraglutide and other anti‐diabetic agents such as thiazolidines would exert beneficial effects on the metabolism of our 12‐week‐old mouse model.

Enhanced pancreatic β‐cell apoptosis leads to β‐cell loss in T2DM, which is characterized by progressive deterioration and ultimate failure of β‐cell function 42. The present study revealed increased apoptosis of pancreatic islet β‐cells in diabetic mice accompanied by a significant decrease of islet size. However, treatment of 12‐week‐old diabetic mice with liraglutide resulted in decreased islet β‐cell apoptosis and increased islet size. Experimental evidence indicated that GLP‐1 receptor agonists enhance β‐cell proliferation and prevent β‐cell apoptosis. 18, 24, 26, 43. Moreover, it was reported that liraglutide did not affect islet endocrine non‐β‐cells. Αlpha‐cell (glucagon‐positive cell) and delta‐cell (somatostatin‐positive cell) mass was not changed by chronic liraglutide treatment of alloxan‐induced diabetic mice and diabetic ZDF rats 44, 45. Therefore, besides the well‐documented positive effect of liraglutide on the proliferation of β‐cells, our data suggest that liraglutide increases islet size, at least in part, by preventing further apoptotic loss of β‐cells. However, after 2 weeks of treatment, liraglutide did not manage to restore islet β‐cell insulin content in diabetic animals.

We have previously established a pivotal role for nephrin in pancreatic β‐cell survival signalling, substantiating its anti‐apoptotic function 32. Signalling downstream of nephrin involves PI3‐kinase‐mediated AKT activation, which in turn promotes cell survival by inhibiting apoptosis *via* phosphorylation and inactivation of pro‐apoptotic targets including BAD and FoxO 32. Studies in pancreatic islets of diabetic mice revealed an *in vivo* reduction of nephrin expression. Gradual reduction of nephrin expression in long‐standing hyperglycaemia may further compromise islet β‐cell survival. In fact, we have previously reported that nephrin silencing increases susceptibility of cultured pancreatic β‐cells to apoptosis 32. Our results demonstrated that treatment of diabetic mice with liraglutide resulted in increased expression of nephrin. Liraglutide‐induced nephrin expression could thus contribute, at least in part, to improved islet β‐cell survival or reduced apoptosis. The mechanism of liraglutide action on nephrin expression awaits further investigation.

The molecular mechanism of β‐cell protection by liraglutide was examined in a mouse pancreatic β‐cell line, βTC‐6 cells, which have been shown to express GLP‐1R 46. In competition assays, GLP‐1R specifically bound GLP‐1 analogues such as extendin‐4, which in turn enhanced glucose‐stimulated insulin secretion from βTC‐6 cells 46. Our finding that liraglutide inhibited apoptosis in serum‐deprived βTC‐6 cells indicates a direct anti‐apoptotic effect of GLP‐1 analogue transduced *via* the GLP‐1 receptor. According to our results, βTC‐6 cell protection by liraglutide is elicited *via* inhibition of caspase‐3 activation. Considering that activated caspase‐3 signalling is involved in β‐cell apoptosis in islets of animals and humans with T2DM 42, liraglutide may well reflect the action of GLP‐1, as GLP‐1R activation leads to inhibition of caspase activation and increased survival of β‐cells 17, 47.

Findings from our study in βTC‐6 cells indicated that liraglutide induces PI3K‐dependent AKT phosphorylation. AKT activation is accompanied by phosphorylation, hence inhibition, of pro‐apoptotic BAD protein and suppression of FoxO1/3a transcription factors, known to be important for pancreatic β‐cell survival 48. In support of our findings, the GLP‐1R agonist, exendin‐4 decreased activation of caspase‐3 and prevents β‐cell apoptosis through AKT/PKB and MAPK signalling in db/db diabetic mice 49. AKT promotes cell survival by direct phosphorylation of BAD, a member of the Bcl‐2 family 50. According to our results, activated AKT mediates the Ser‐136 phosphorylation of BAD. Ser‐136 phosphorylation of BAD appears to be the most predominant 14‐3‐3‐binding site 51. It has been reported that in the absence of insulinotropic glucose concentration, activation of GLP‐1R by GLP‐1 in pancreatic β‐cells stimulates phosphorylation of BAD at Ser‐112, through the β‐arrestin1‐dependent ERK1/2‐p90RSK signalling 52. Phosphorylation of BAD at Ser‐136 or Ser 112 favours binding of BAD to the scaffold protein 14‐3‐3 in the cytoplasm, preventing the interaction of BAD with Bcl‐xL and Bcl‐2 at mitochondrial membranes, thus inhibiting apoptosis 51, 52.

FoxO transcriptional activity is negatively regulated through phosphorylation‐dependent nuclear exclusion of FoxO, which is mediated by PI3K/AKT and epidermal growth factor receptor (EGFR) signalling 53. FoxO1 factor mediates GLP‐1 effects on β‐cell proliferation and survival. The anti‐apoptotic effects of GLP‐1 in β‐cells are mediated by promotion of FoxO1 phosphorylation‐dependent nuclear exclusion and consequent up‐regulation of PDX‐1 and Foxa2 expression *via* EGFR‐ and PI3K‐dependent activation of AKT 54, 55. Our results demonstrated that the GLP‐1 analogue liraglutide induced phosphorylation, thus inhibition of FoxO1/3, *via* PI3‐kinase‐dependent AKT activation and further suggest that at the level of cellular signalling, liraglutide may mimic the mode of action of GLP‐1. Inhibition of FoxO1 was reported to mediate the proliferative and pro‐survival effects of exendin‐4, another GLP‐1 agonist, in insulinoma cells and transgenic mice 55. Conversely, the ability of exendin‐4 to increase β‐cell mass was suppressed in transgenic mice constitutively expressing nuclear FoxO1 in β‐cells 55.

In conclusion, this study demonstrates that the human long‐lasting GLP‐1 analogue, liraglutide, independent of effects on metabolic control in the db/db mouse model of degenerative diabetes, restores β‐cell mass in diabetic mice, at least in part, *via* inhibition of islet β‐cell apoptosis. According to our findings, restoration of nephrin expression may also contribute to the enhanced islet β‐cell survival and reduced apoptosis observed in liraglutide‐treated diabetic mice. Our results indicate that protection of β‐cell survival by liraglutide involved inhibition of caspase‐3 activation. The molecular mechanism of the anti‐apoptotic action of liraglutide in β‐cells involves activation of PI3K‐dependent AKT signalling leading to the inactivation of the pro‐apoptotic protein BAD and inhibition of FoxO1 transcription factor. Preservation of a functional β‐cell mass is pivotal for the future therapy of T2DM, aiming at protecting β‐cells from apoptotic cell death. Therefore, advanced knowledge of diabetes pathophysiology and a better understanding of the mechanism of GLP‐1 analogues' action on β‐cell signalling and function will contribute to the development of novel therapeutics in the treatment of T2DM.

## Conflicts of interest

The authors confirm that there are no conflicts of interest.
